# Hypoxia induces expression of angiotensin‐converting enzyme II in alveolar epithelial cells: Implications for the pathogenesis of acute lung injury in COVID‐19

**DOI:** 10.14814/phy2.14854

**Published:** 2021-05-15

**Authors:** Anne Sturrock, Elizabeth Zimmerman, My Helms, Theodore G. Liou, Robert Paine

**Affiliations:** ^1^ Department of Veterans Affairs Medical Center Salt Lake City Utah USA; ^2^ Division of Respiratory Critical Care and Occupational Pulmonary Medicine University of Utah School of Medicine Salt Lake City Utah USA

## Abstract

SARS‐CoV‐2 uptake by lung epithelial cells is a critical step in the pathogenesis of COVID‐19. Viral entry is dependent on the binding of the viral spike protein to the angiotensin converting enzyme II protein (ACE2) on the host cell surface, followed by proteolytic cleavage by a host serine protease such as TMPRSS2. Infection of alveolar epithelial cells (AEC) in the distal lung is a key feature in progression to the acute respiratory distress syndrome (ARDS). We hypothesized that AEC expression of ACE2 is induced by hypoxia. In a murine model of hypoxic stress (12% FiO2), the total lung Ace2 mRNA and protein expression was significantly increased after 24 hours in hypoxia compared to normoxia (21% FiO2). In experiments with primary murine type II AEC, we found that exposure to hypoxia either in vivo (prior to isolation) or in vitro resulted in greatly increased AEC expression of both Ace2 (mRNA and protein) and of Tmprss2. However, when isolated type II AEC were maintained in culture over 5 days, with loss of type II cell characteristics and induction of type I cell features, Ace2 expression was greatly reduced, suggesting that this expression was a feature of only this subset of AEC. Finally, in primary human small airway epithelial cells (SAEC), ACE2 mRNA and protein expression were also induced by hypoxia, as was binding to purified spike protein. Hypoxia‐induced increase in ACE2 expression in type II AEC may provide an explanation of the extended temporal course of human patients who develop ARDS in COVID‐19.

## Graphical abstract

This figure shows relative ACE2 mRNA expression in lung homogenate and isolated type II alveolar epithelial cells from mice exposed to a hypoxic environment compared to control mice in normoxia. Hypoxic exposure in vivo results in significant increased expression of type II cell ACE2 and TMPRSS2. These data suggest a mechanism by which hypoxia facilitates entry of SARS‐CoV‐2 into alveolar epithelial cells.

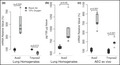

## 1 INTRODUCTION

Binding and internalization of SARS‐CoV‐2 virus by respiratory epithelial cells is a critical step in the pathogenesis of COVID‐19, the clinical disease due to this novel corona virus. Viral entry is dependent on binding of the SARS‐CoV‐2 spike protein to the angiotensin converting enzyme II protein (ACE2 in humans, Ace2 in mice) on the host cell surface, following by proteolytic cleavage by a host serine protease, such as Transmembrane protease serine 2 (TMPRSS2 in human, Tmprss2 in mice) (Aguiar et al., 2020; Hoffmann et al., 2020; Shang et al., 2020). While viral infection of upper airway cells may result in symptomatic disease and viral shedding, it is likely that infection of alveolar epithelial cells (AEC) in the distal lung drives progression to severe lung injury and the acute respiratory distress syndrome (ARDS) (Dinnon et al., 2020). The regulation of ACE2 expression on AEC and its implications for viral infection in the alveolar space are poorly understood. Type II AEC are the source of pulmonary surfactant and serve as stem cells to help repopulate the alveolar surface following lung injury. Although numerically representing approximately 50% of the cells of the alveolar epithelium, type II AEC cover less than 10% of the alveolar surface; the rest of the alveolar surface is covered by large, thin type I AEC (Crapo et al., 1983). We hypothesized that AEC expression of ACE2 and TMPRSS2 are upregulated by hypoxia. Furthermore, we examined the relationship of ACE2 expression to expression of features of the type II AEC phenotype. To explore this hypothesis, we first used a murine model of hypoxia, allowing us to assess Ace2 and Tmprss2 expression at the level of the whole lung and specifically in type II AEC ex vivo. Subsequent studies extend this work to human primary small airway epithelial cells (SAEC). We found that expression of ACE2 and TMPRSS2 are induced in the setting of hypoxic stress in vivo and in vitro and that expression of these molecules is associated with expression of type II cell characteristics. We believe that this work offers important insights to explain the distinctive temporal course and reports of distinctive physiology of human patients who develop ARDS in COVID‐19.

## 2 METHODS

### 2.1 Animals, lung homogenates and AEC *ex vivo* and culture

Animals: Wild‐type (WT) C57BL/6 (Ly5.1; CD45.2) mice were obtained from Jackson Laboratory (Bar Harbor, ME). Both male and female mice, aged 6–12 weeks, were used in these experiments. Mice were housed under specific pathogen‐free conditions and were monitored daily by veterinary staff. Mice were exposed to hypoxic conditions (12% oxygen) for 48 h or maintained in normoxia (21% oxygen), then euthanized and the lungs immediately harvested for RNA extraction or AEC isolation, as in our prior work (Sturrock et al., 2018). The animal care committee at the Salt Lake City VA Medical Center approved these experiments.

Lung homogenates: Lungs were snap frozen in dry ice/ methanol, homogenized in RNA extraction buffer from a *Quick*‐RNA MiniPrep (Zymed) and cleared by centrifugation.

Ex vivo and cultured mouse primary AEC: AEC were isolated from mice exposed to 21% or 12% oxygen for 48 h, as in our prior work (Sturrock et al., 2018). Type II AEC were initially cultured in HITES medium +10% fetal bovine serum (FBS) in ambient air with 5% CO_2_ (normoxia) for 48 h. The medium was then replaced with fresh medium and the cultures were placed in normoxia or hypoxia (1% oxygen, 5% CO2) for 24 h (Sturrock et al., 2018). In select experiments, cells were treated with the proline hydrolase inhibitor, dimethyloxalylglycine (DMOG) to chemically stabilize hypoxia inducible factor (HIF)‐1α (Milkiewicz et al., 2004).

### 2.2 Human Small airway epithelial cells (SAEC)

Human SAEC were purchased from Lonza Bioscience and maintained in complete small airway growth medium (Lonza). All experiments were performed on p4 cultures and on cells from three separate donors.

### 2.3 RNA preparation and Real‐time (RT)‐PCR

Total cellular RNA was isolated using the *Quick*‐RNA MiniPrep (Zymed); first strand cDNA was reverse transcribed using HighCapacity kit (ABI) and specific amplification carried out in a StepOnePlus (ABI). Gene‐specific primers were designed using the Roche Applied Science Universal Probe Library Assay Design Center. Biological samples were amplified in duplicate, which were averaged to form a single data point. A constant amount of total RNA (1 µg) was converted to cDNA for RT‐PCR. As previously reported, a number of “housekeeping” genes are altered in hypoxia (Sturrock et al., 2018). For these experiments, we used pol2A as a reference gene due to its limited response to changes in oxygen tension. Results are expressed as negative log‐fold change compared to normoxic control.

### 2.4 ACE2 proteins

ACE2 ELISAs. Whole cell extracts (EpiQuik Whole Cell Extraction Kit; EpiGentek) were prepared from murine AEC or human SAEC maintained in either 21% or 1% oxygen for 24 h. ACE2 levels were determined in 20 µg of protein (BCA assay; Pierce) using a murine and human ACE2 Duoset ELISAs from R&D Systems.

Binding to SARS‐CoV‐2 spike protein. The amount of functional ACE2 in SAEC extracts (100 µg protein) able to bind to SARS‐CoV‐2 spike protein was determined using a CoviDrop™ SARS‐CoV‐2 Spike‐ACE2 Binding Activity/Inhibition Assay Kit (EpiGentek). A standard curve generated using a supplied assay standard allowed for quantification.

### 2.5 Statistical analysis

We used the R statistical system (R Core Team 2020) for statistical analyses and visualization. We used *t*‐tests and multivariable linear regression to compare single or multivariable data, respectively, with controls. We used linear regression to analyze the relationships between mRNA expression as the dependent variable and day in culture as the independent variable. We calculated fold‐change for each sample (*FCsample*) after measuring the fractional PCR doubling cycles required so that SYBR Green fluorescence exceeded the threshold for detection (*C_T_*). We used the following formula:

$$FCsample=2^{‐(\Delta C_{T}sample‐\Delta C_{T}median)},$$

where *ΔC_T_sample* was the number of doubling cycles to detect each mRNA minus the number of doubling cycles to detect mRNA from the reference gene for each sample, and the *ΔC_T_median* was the median *ΔC_T_sample* for control samples. In the figures, the relative value of mRNA expression is shown as the negative base 2 log transformed values. We considered *p* < 0.05 as significant and displayed data as box plots (McGill & JW, Larsen, WA., 1978).

## 3 RESULTS

### 3.1 Impact of in vivo hypoxia

**FIGURE 1:**
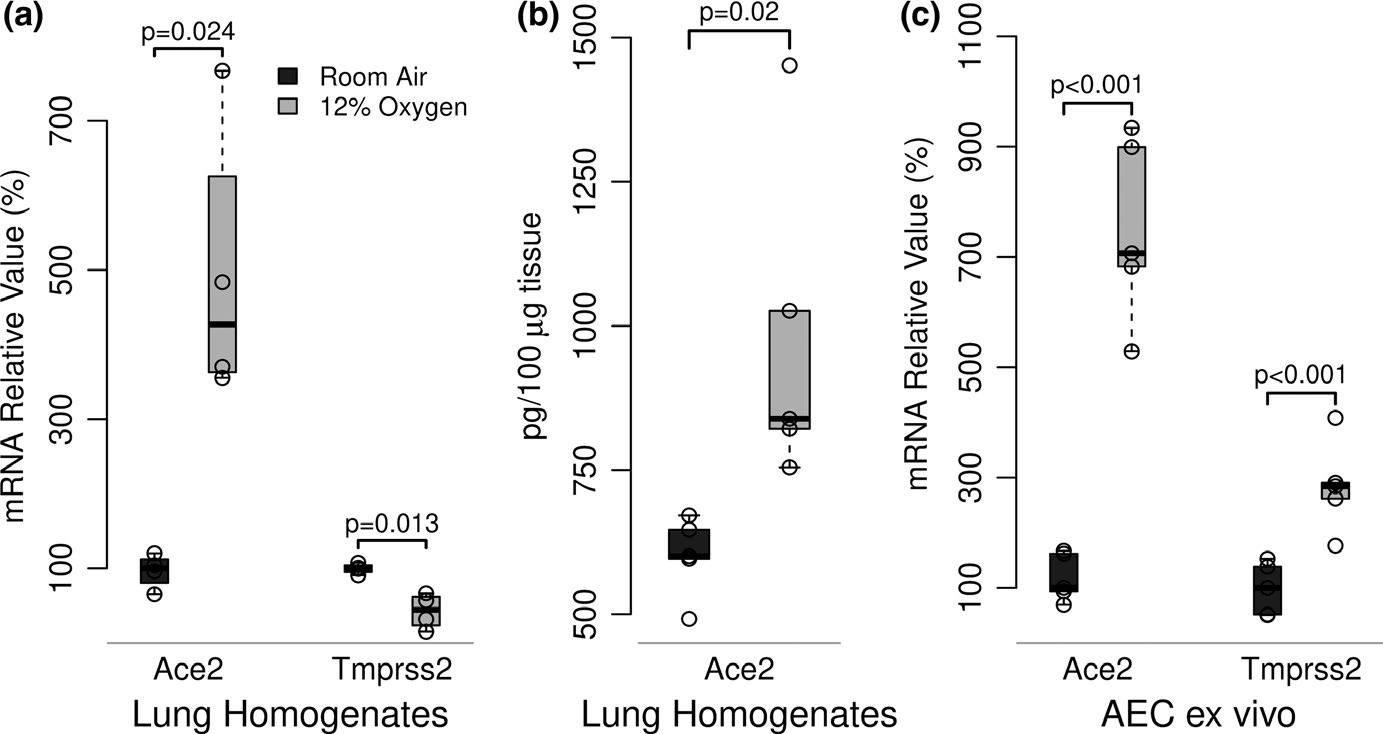
*Effect of in vivo hypoxic exposure on lung ACE2 and TMPRSS2 expression*. Mice were exposed to room air (21% oxygen) or hypoxia (12% oxygen) for 48 h. Relative mRNA expression in whole lung homogenates (Figure 1a) or in type II AEC ex vivo (Figure 1c) was determined by RT‐PCR and are expressed relative to the normoxic control. Ace2 protein expression in lung homogenates was determined by ELISA (Figure 1b). Boxplots (McGill et al., 1978) show data representative of two independent (n = 3) experiments; *p*‐values show differences between paired boxplots

We first determined the effect of exposure of mice to a hypoxic environment in vivo on expression of Ace2 in lung homogenates. After 24 h in 12% oxygen, lung Ace2 mRNA expression was increased almost 5‐fold (Figure 1a). At the same time, there was a modest reduction of Tmprss2 mRNA expression. Ace2 protein expression was also increased significantly (Figure 1b). To determine the effect of hypoxia specifically on type II AEC expression of Ace2 and Tmprss2, type II AEC were isolated from mice after 24 h in hypoxia or from normoxic controls. RNA was extracted immediately after cell isolation. Type II AEC Ace2 expression was dramatically increased in cells from mice in hypoxic conditions compared to cells from mice in normoxia (Figure 1c). In addition, Tmprss2 expression increased in type II AEC from mice in hypoxia (Figure 1c). These data provide a mechanism by which SARS‐CoV‐2 binding and internalization by epithelial cells in the alveolar space would be enhanced during hypoxic stress.

### 3.2 Impact of hypoxia on Ace2 expression by murine AEC in vitro

**FIGURE 2:**
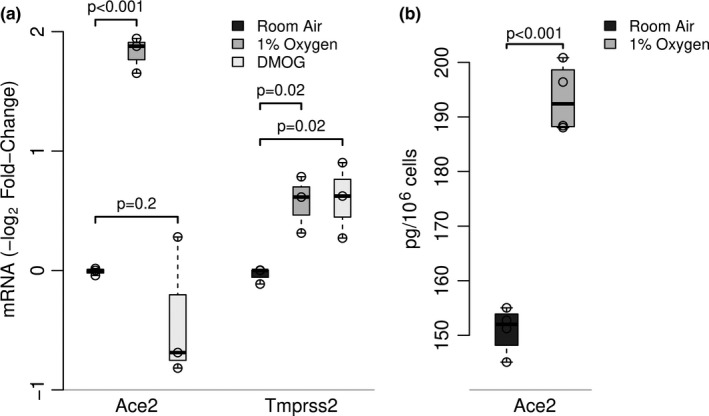
*Effect of in vitro hypoxic exposure in vitro on mouse primary AEC expression of ACE2 and TMPRSS2*. AEC cultures were exposed to 1% oxygen, 21% oxygen, or DMOG (1 µM, in 21% oxygen) for 24 h. Relative mRNA expression of Ace2 and Tmprss2 was determined by RT‐PCR and expressed relative to the normoxic control (Figure 2a). Ace2 protein expression was determined in AEC extracts by ELISA (Figure 2b). Boxplots (McGill et al., 1978) show data representative of 3 independent (n = 3) experiments; *p*‐values from multivariable linear regressions are from comparisons with control conditions

We next explored the effect of in vitro hypoxic stress on Ace2 expression by primary murine AEC. Type II AEC were allowed to adhere in culture for 48 h, then placed in hypoxic (1% oxygen) or normoxic (21% oxygen) conditions for 24 h. Ace2 mRNA (Figure 2a) and protein (Figure 2b) expression were increased following exposure to hypoxic conditions compared to normoxic cells. Many effects of hypoxic stress are mediated by HIF stabilization and downstream transcription of HIF‐responsive genes. We have shown previously that 24 h exposure of AEC to 1% oxygen leads to induction of HIF‐responsive genes, such as HO‐1, VEGF, and NOS (Sturrock et al., 2018). We have also found previously that treatment of AEC with DMOG results in HIF‐1 stabilization and induction of these HIF‐target genes (Sturrock et al., 2018). However, DMOG failed to induce Ace2 mRNA expression in AEC (Figure 2a). Conversely, both hypoxia and DMOG induced modest increases Tmprss2 expression. Thus, the induction of Ace2 expression in AEC observed following in vivo hypoxia is, to a significant degree, a direct response of type II AEC to hypoxia itself. Furthermore, HIF‐1 stabilization is not sufficient to induce increased Ace2 expression.

### 3.3 Relationship of Ace2 expression to AEC characteristics in vitro

**FIGURE 3:**
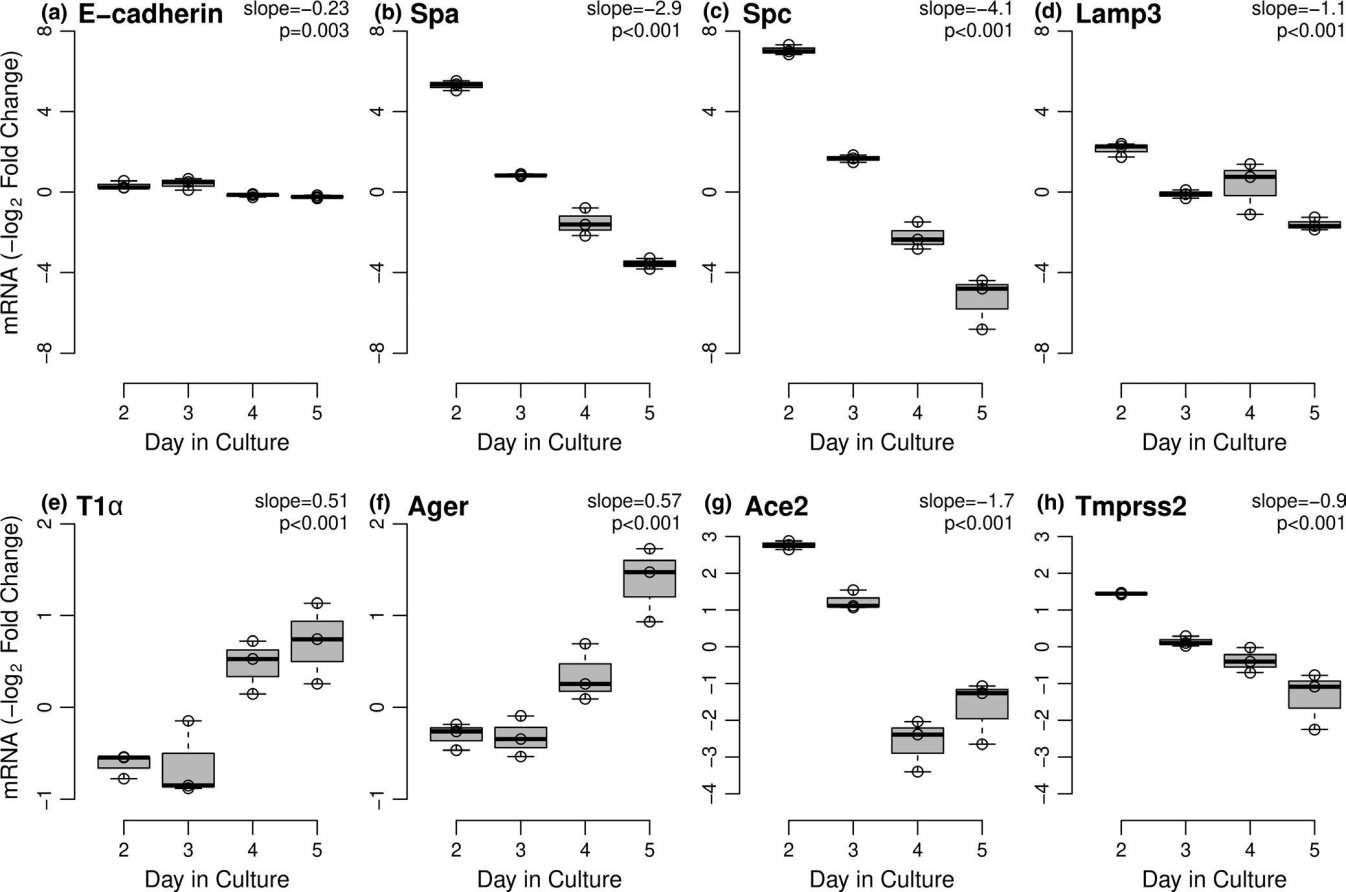
*Changes in expression of AEC phenotypic markers*, *Ace2 and Tmprss2 over time in culture*. Relative mRNA expression of the pan‐epithelial marker, E‐cadherin (Figure 3a) and primary murine AEC phenotypic markers for type II (Figure 3b‐d) and type I (Figure 3e‐f) AEC was determined by RT‐PCR in cells maintained in 21% oxygen over 5 days in culture. Expression of Ace2 and Tmprss2 mRNA are shown in Figure 3g, h, respectively. Boxplots (McGill et al., 1978) show data representative of two independent (n = 3) experiments. Linear regression slopes and *p*‐values are shown with each transcript

Expression of AEC phenotypic features changes in a characteristic pattern when murine type II AEC are placed in cell culture on tissue culture plastic (Reynolds et al., 2010; Mir‐Kasimov et al., 2012). Although the cells continue to express E‐cadherin, expression of many typical type II AEC features, including the surfactant proteins, SpA, SpC, and Lamp3 decrease greatly over time (Figure 3b‐d). Conversely, some characteristics of type I AEC, such as expression of RAGE and T1α, are induced over this same period (Figure 3e,f). Interestingly, expression of Ace2 decreased with time in culture as the cells lose features of the fully differentiated type II cell phenotype (Figure 3g). Expression of Tmprss2 also decreased significantly over time (Figure 3h), although to a lesser extent than that of Ace2. These data suggest that Ace2 expression may be associated more closely with the type II AEC phenotype in the alveolar epithelium.

### 3.4 Impact of hypoxia on ACE2 expression by human SAEC

**FIGURE 4:**
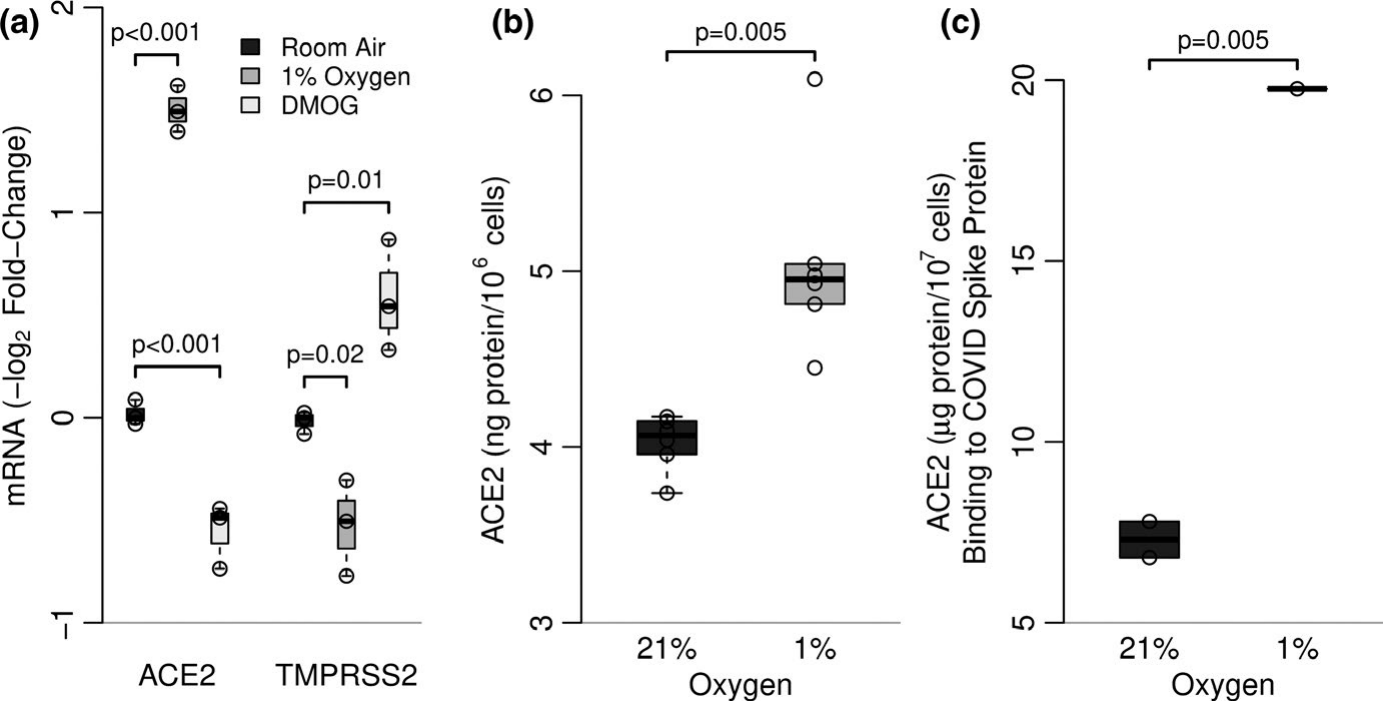
*Effect of hypoxia exposure on expression of ACE2 in human SAEC*. Relative mRNA expression for ACE2 and TMPRSS2 was determined by RT‐PCR in SAEC exposed to 1% oxygen, 21% oxygen or DMOG (1 µM, in 21% oxygen) for 24 h (Figure 4a). Boxplots (McGill et al., 1978) show data representative of three independent (n = 3) experiments, each using cells derived from a different donor; *p*‐values are from multivariable linear regressions with control conditions. ACE2 protein levels (Figure 4b) were determined by ELISA (R&D Systems) using whole cell extracts of SAEC (10^6^ cells/sample) after 24 h exposure to 1% or 21% oxygen. Boxplots show data representative of two independent (n = 3) experiments; *p*‐values are from *t*‐test comparisons of paired groups. Following 24 h exposure to 1% or 21% oxygen, the amount of ACE2 able to bind immobilized COVID‐19 spike protein was determined in whole cell extracts from SAEC (Figure 4c). Boxplots show data from an average of two samples per condition; the *t*‐test *p*‐value is shown. A similar result was obtained using SAEC cells from a second donor

We evaluated ACE2 mRNA and protein expression in primary human cells from the peripheral lung (SAEC). ACE2 mRNA (Figure 4a) and protein (Figure 4b) were expressed under normoxic conditions in SAEC and expression was increased following 24 h in hypoxic conditions. Similar to primary murine AEC, the effect of hypoxia was not mimicked by chemical stabilization of HIF with DMOG. In addition to measuring ACE2 protein expression by ELISA, we directly assessed SARS‐CoV‐2 spike protein binding to SAEC extracts. Spike protein binding to SAEC extracts was increased by exposure of SAEC to hypoxia (Figure 4c). Thus, primary human epithelial cells from the peripheral lung recapitulate key findings from our murine model.

## 4 DISCUSSION

The emergence of the novel coronavirus causing COVID‐19 has resulted in a devastating pandemic. An important feature of COVID‐19 is its highly variable clinical course, from asymptomatic carriage (Sakurai et al., 2020) to acute respiratory failure and ARDS (Richardson et al., 2020; Zhou et al., 2020). Efforts to understand the factors determining the severity of illness have largely focused on host characteristics, such as age, gender, and comorbidities. Infection is most commonly acquired through inhalation of aerosols or droplets containing viral particles (Lu et al., 2020; Liu et al., 2020; Prather et al., 2020), with initial viral entry into nasopharyneal cells triggering early inflammatory responses (Tay et al., 2020). When severe, viral infection reaches the lower respiratory tract, where the epithelium is injured directly by the virus or due to an overly exuberant inflammatory response. (Li & Lin, 2013).

We now present data concerning the expression of ACE2 by epithelial cells in the peripheral lung. In our murine model, we confirm ACE2 expression by primary type II AEC and show that ACE2 expression is increased significantly following exposure to hypoxic environments in vivo or in vitro. This induction does not appear to be a direct consequence of HIF stabilization (Jahani et al., 2020; Ren et al., 2019; Nizet & Johnson, 2009). Interestingly, within the alveolar space, ACE2 expression is a characteristic of type II AEC and may be lost as cells lose other features of the fully differentiated cell type. We also found that human SAEC in primary culture express ACE2 mRNA and protein (Aguiar et al., 2020) and that ACE2 expression and spike protein binding are induced by exposure to hypoxia. These findings have important implications for SARS‐CoV‐2 pathobiology.

One of the notable characteristics of COVID‐19 is the slow progression from initial onset of disease to acute respiratory failure and ARDS (Richardson et al., 2020; Zhou et al., 2020). Our findings offer insight into this course. First, type II AEC numerically represent approximately half of the population of AEC overall but cover only 7% of the alveolar surface, with the remaining 93% covered by type I AEC (Crapo et al., 1983). Thus, only a limited portion of the alveolar surface is initially susceptible to SARS‐CoV‐2 infection. As individuals become increasingly ill, with ventilation perfusion mismatching leading to regional hypoxia, ACE2 expression is further induced, with increased opportunity for viral entry into type II AEC. As injury progresses, with loss of AEC due to either viral infection per se or the inflammatory process, the alveolar epithelium is repopulated with new type II cells. These new type II AEC also express ACE2 and provide new targets for viral entry. This pattern may explain the prolonged course of respiratory failure in patients with ARDS due to COVID‐19.

Hypoxia has multiple important effects on susceptibility to infection in the peripheral lung. Previously we have shown decreased expression of key innate immune molecules by AEC that have been exposed to hypoxia (Sturrock et al., 2018). Of particular importance is granulocyte macrophage colony‐stimulating factor (GM‐CSF), a growth factor whose presence in the lung is required for normal alveolar macrophage functional maturation (Trapnell & Whitsett, 2002). GM‐CSF expression by AEC is greatly suppressed by exposure to low oxygen environments both in vivo and in vitro (Sturrock et al., 2018). Thus, local hypoxia may simultaneously increase alveolar targets for viral entry while disrupting normal host defense networks.

There are several important features of our experimental approach. Mouse models offer powerful experimental approaches to understand the pathobiology of lung injury. The lung is a complex organ composed of many different types of cells. By isolating AEC from the lungs of mice exposed to hypoxic conditions, we are able to distinguish the specific effects on expression by AEC of the molecules of interest. We also confirmed that the effects on Ace2 expression are a direct consequence of the hypoxic stress on these epithelial cells. Because the SARS‐CoV‐2 virus that causes COVID‐19 does not infect mice (Dinnon et al., 2020), we confirmed our findings in primary human cells from the peripheral lung (SAEC).

There are several limitations to this study. We postulate that the loss of Ace2 expression as murine AEC lose type II cell features in culture reflects a close association between Ace2 expression and type II AEC‐specific differentiation. Additional studies will be required to elucidate the details of the mechanisms relating Ace2 expression during epithelial cell differentiation. Furthermore, the specific mechanism driving increased Ace2 expression in the setting of hypoxia is not yet known. Our data suggest that this induction is not a direct effect of HIF stabilization. We speculate that it may be a part of a stress response by AEC, perhaps related to potentially beneficial effects of ACE2 binding by its endogenous ligands.

## 5 CONCLUSION

These studies demonstrate that expression of ACE2 and TMPRSS2 by primary cells in the gas exchange regions of the lung are induced by hypoxia. Our data also suggest that ACE2 is preferentially expressed on type II AEC and that expression of ACE2 is lost as ACE loose features of the type II cell phenotype. These observations have important implications for the pathophysiology of SARS‐CoV‐2 infection and the clinical course of COVID‐19.

## DISCLOSURES

The authors have no conflicts to declare related to this work. In activities unrelated to the current work, RP serves as a consultant to Partner Therapeutics.

## AUTHOR CONTRIBUTIONS

RP and AS conceived of the project and designed the experiments. Experiments were carried out by AS and EZ. MY and TL participated in data analysis and presentation. The manuscript was drafted by AS and RP. All authors participated in final editing and approved of the manuscript as submitted.
